# Biosimilar filgrastim treatment patterns and prevention of febrile neutropenia: a prospective multicentre study in France in patients with solid tumours (the ZOHé study)

**DOI:** 10.1186/s12885-018-4986-1

**Published:** 2018-11-16

**Authors:** Henri Roché, Jean-Christophe Eymard, Abderraouf Radji, Alain Prevost, Rafik Diab, Michele Lamuraglia, Ravaka-Fatoma Soumoudronga, Isabelle Gasnereau, Alain Toledano

**Affiliations:** 1 Institut Claudius Regaud, Institut Universitaire du Cancer Toulouse – Oncopole, 1 avenue Irène Joliot-Curie, 31059 Toulouse Cedex 9, France; 2 Institut Jean Godinot, Reims, France; 3 Centre Frédéric Joliot, Rouen, France; 4 Centre Médical Spécialisé de Praz-Coutant, Passy, Paris, France; 5 Assistance Publique Hôpitaux de Paris – Hôpital Beaujon, Clichy, Paris, France; 6 Sandoz France, Levallois-Perret, France; 7 Institut de Cancerologie Hartmann, Levallois-Perret, France

**Keywords:** Biosimilar pharmaceuticals, Filgrastim, Febrile neutropenia, Real-world, Chemotherapy, Solid tumours, Guidelines recommendations, Observational study

## Abstract

**Background:**

The ZOHé study was a prospective, non-interventional, multicentre study in France to assess the use of biosimilar filgrastim Zarzio® (Sandoz filgrastim) in routine clinical practice in patients at risk of neutropenia-inducing chemotherapy (CT).

**Methods:**

Patients ≥ 18 years undergoing CT for a malignant disease and with a first prescription for Zarzio® were enrolled in two cohorts according to tumour type: solid tumour or haematological malignancy; results from the solid tumour cohort are reported here. Analyses primarily described the prescription and use of Zarzio® in current practice, and also included identification of factors linked to prescription for primary prophylaxis and comparison of Zarzio® use in relation to European Organisation for Research and Treatment of Cancer (EORTC) guidelines.

**Results:**

Responses were obtained from 125 physicians and 1179 patients with solid tumours, allowing robust statistical analysis of the data. Use of Zarzio® in clinical practice was relatively standardised and followed label indication. The patient profile was in line with EORTC guidelines for granulocyte colony-stimulating factor (G-CSF) febrile neutropenia (FN) prophylaxis, and the majority of patients had ≥ 1 EORTC factor(s) for increased risk of febrile neutropenia. Some patients (10.8%) received Zarzio® despite receiving CT regimens categorised in guidelines as low (< 10%) FN risk (‘over prophylaxis’). Nearly half of patients’ CT regimens did not have a recommended FN risk category. Zarzio® was commonly initiated as primary prophylaxis; initiation in Cycle ≥ 2 of the current line of CT was associated more with a history of neutropenia. The safety profile of Zarzio® was confirmed.

**Conclusions:**

Use of Zarzio® in routine clinical practice is generally in line with EORTC guidelines for prophylaxis of CT-induced neutropenia. Patient-related risk factors appear to be a stronger driver of clinicians’ decision to initiate Zarzio® than CT risk category for FN. The intrinsic risk of FN associated with a specific CT protocol is often miscategorised by physicians. In contrast to earlier reports of underuse of G-CSF prophylaxis, over prophylaxis is observed in a small subgroup of patients with FN risk of < 10%.

**Electronic supplementary material:**

The online version of this article (10.1186/s12885-018-4986-1) contains supplementary material, which is available to authorized users.

## Background

Febrile neutropenia (FN) is a common and sometimes serious complication of myelosuppressive cytotoxic chemotherapy (CT) that can decrease the dose intensity by delaying or reducing the dosage or duration of CT regimens, and thereby compromise the anticancer treatment [1–3]. FN can lead to potentially serious infections that require antibiotic treatment and usually necessitates hospitalisation [4], with a mortality rate of around 8% in the inpatient setting [5].

Granulocyte colony-stimulating factor (G-CSF), a cytokine that stimulates the formation and function of mature neutrophils [6], can be given as prophylaxis to reduce the incidence and duration of FN, thereby decreasing the need to delay or reduce the CT cycles or dose [7–9]. Guidelines in Europe (European Organisation for Research and Treatment of Cancer [EORTC] [10], European Society for Medical Oncology [ESMO] [11]), the USA (National Comprehensive Cancer Network [NCCN] Clinical Practice Guidelines in Oncology v.1.2016 [12], and American Society of Clinical Oncology [ASCO] [13]) recommend prophylactic G-CSF for CT regimens associated with a high risk of FN (greater than 20%) and consideration of G-CSF in patients at intermediate FN risk (10–20%), particularly if additional risk factors such as advanced age, advanced disease, previous episodes of FN or certain comorbidities are present.

The first recombinant human G-CSF, filgrastim, entered into clinical practice in the 1990s and biosimilar versions have been available in Europe since 2008. Biosimilars are successors to biological medicines that have lost patent exclusivity, which match the reference molecule in structural and functional characteristics such that clinicians and patients can expect the same safety and efficacy. Regulatory approval is based on the totality of evidence for quality, safety and efficacy data demonstrating that there are no clinically meaningful differences between the reference molecule and the biosimilar (European Medicines Agency Guideline on similar biological medical products [14]). Benefits of G-CSF biosimilars over the original molecule are mainly cost reductions, as equivalent clinical safety and efficacy, as part of the totality of evidence, was demonstrated in pharmacodynamic studies and confirmed by observational data [15, 16]. Having confirmed bioequivalence in phase I and phase III studies [17, 18], the biosimilar Sandoz filgrastim was approved in February 2009 for the same indications as the reference product (Neupogen®) [19, 20], including myelosuppressive CT-induced neutropenia [17], and marketed under the name Zarzio® in Europe. There are extensive long-term data on its safety and efficacy in clinical practice [21–23]. Zarzio® was the first biosimilar to receive marketing approval in the USA, in March 2015, under the name Zarxio® [24].

While earlier studies found that G-CSF prophylaxis for CT-induced neutropenia was underused in real-world practice in Europe [25, 26], more recent studies suggest that the widespread availability and reduced cost of biosimilar G-CSFs are leading to increased use [21, 27]. Widespread acceptance of biosimilar filgrastim to prevent CT-induced neutropenia and maintain CT dose is reflected in the current EORTC guidelines [10]; however, numerous agents and new CT combinations have been brought into routine practice since the last update. The ZOHé study aimed to evaluate the use of biosimilar Zarzio® by oncologists and haematologists as primary or secondary prophylaxis in routine practice in patients receiving CT for solid tumours (ST) or haematological malignancies (HM). This paper reports the results for the cohort of patients with ST.

## Methods

### Study design and patients

The study design and characteristics of patients participating in the ZOHé study have previously been reported [28]. In brief, the use of biosimilar filgrastim (Zarzio®, Sandoz, Holzkirchen, Germany) was evaluated in a total of 1816 patients (1179 with ST) in routine clinical practice at 125 sites in France between June 2013 and April 2014.

Patients aged 18 years or older undergoing cytotoxic CT for ST or HM who had a first prescription for Zarzio® were included. Patients already in an ongoing interventional study involving Zarzio® treatment were excluded. Data from the ST patient cohort of ZOHé are reported here.

### Data collection

Patients underwent an inclusion visit (V1) on the first day of the CT cycle during which Zarzio® was initiated, and a follow-up visit (V2) 3 months after inclusion or on discontinuation of CT or Zarzio® (if this occurred before 3 months after inclusion). Baseline patient characteristics were collected at V1, and data on the use of Zarzio®, patient clinical status, occurrence of neutropenia and adverse events (AEs) at V2.

Data were collected using an electronic case report form and monitored centrally for quality control; AEs were coded using version 17.0 of the Medical Dictionary for Regulatory Activities (MedDRA). Obligatory fields, drop-down lists and consistency controls verified immediately on data entry were set up to limit missing or incoherent data.

### Analyses

The primary objective was to describe indications and use of Zarzio® in routine clinical practice in patients receiving CT for ST or HM. Other aims were to describe the characteristics of patients treated with Zarzio®, characterise the subgroup of patients aged over 70 years (data to be reported elsewhere) and to identify factors associated with the implementation of primary prophylaxis for FN (defined as G-CSF prophylaxis introduced during the first cycle of the current CT regimen).

### Statistical methodology

This manuscript focusses on patients with solid tumours who participated in the ZOHé study. Statistical methods have previously been described in a publication specifically describing the outcomes for patients with haematological malignancies [28]. Briefly, descriptive statistics were applied using a 95% confidence interval. Imputation of missing data was not carried out for the analyses.

## Results

### Study population

**Fig. 1:**
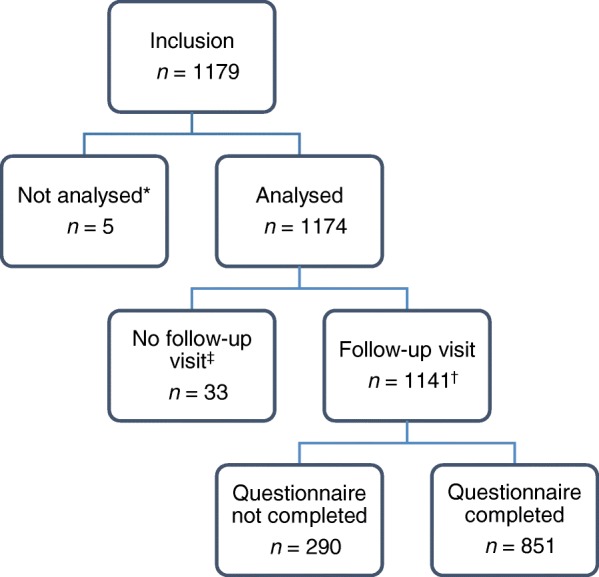
Study population for the ST cohort. *Five patients were excluded from the analysis: four due to deviation from the eligibility criteria and one because of a duplicated patient record. ^†^Of these patients, 261 left the study before the 3-month follow-up visit but were reviewed for data collection. ^‡^Patients who left the study prematurely and were not followed up were comparable with the followed-up patients in terms of age, sex, tumour type and stage, comorbidities, and CT treatments. They had significantly more prior episodes of neutropenia (*p*=0.04) and a worse performance score (*p*<0.01) at inclusion compared with the followed-up patients. *CT* chemotherapy, *ST* solid tumour

Patients were recruited by a representative sample of 60 oncologists, 35 haematologists and 30 other specialties from 125 hospitals in France. Of the 1807 patients included in the ZOHé analysis population, 1174 (65%) received CT for ST (Fig. 1). Data from the follow-up visit were collected for 1141 patients in the ST cohort; reasons for patients leaving the study before the 3-month endpoint are available in Additional file 1: Table S1.

### Patient demographics and clinical characteristics of the ST cohort

Baseline patient characteristics are shown in Table 1. The most common tumour types were breast, lung and gastrointestinal (GI). The mean age was 62.3 ± 12.0 years, but varied for the different tumour types, with the breast cancer subgroup being younger (57.7 ± 11.9 years) and the prostate cancer subgroup being older (71.7 ± 7.2 years), in keeping with the known demographics of these cancers. A quarter of patients had comorbidities or pathologies at risk of FN.

**Table 1:** Patient characteristics of the ST cohort at inclusion (*N* = 1174)*

Characteristic	Breast	Lung	GI: colorectal	GI: non-colorectal	GYN: ovarian	GYN: non-ovarian	Head & neck	Prostate	Other^§^	Total
*n* (% of total)	356 (30.3)	280 (23.9)	103 (8.8)	69 (5.9)	72 (6.3)	39 (3.3)	104 (8.9)	66 (5.6)	81 (6.9)	1174
Age, mean ± SD (years)	57.7 ± 11.9	63.9 ± 10.4	66.4 ± 11.4	63.9 ± 10.0	65.7 ± 11.6	66.2 ± 10.9	59.7 ± 11.0	71.7 ± 7.2	61.0 ± 15.6	62.3 ± 12
≥ 70 years, *n* (%)	57 (16.0)	76 (27.1)	37 (35.9)	22 (30.6)	30 (41.7)	18 (46.2)	16 (15.2)	37 (56.1)	29 (35.8)	322 (27.4)
Female, *n* (%)	351 (98.6)	87 (31.1)	45 (43.7)	26 (36.1)	72 (100)	39 (100)	15 (14.3)	0 (0)	12 (14.8)	647 (55.1)
ECOG PS, *n* (%)	*n* = 354	*n* = 277	*n* = 100	*n* = 71	*n* = 68	*n* = 36	*n* = 104	*n* = 64	*n* = 79	*n* = 1153
Grade 0–1	320 (90.4)	203 (73.3)	92 (92.0)	50 (70.4)	52 (76.5)	32 (88.9)	90 (86.5)	42 (65.6)	59 (74.7)	940 (81.5)
Grade ≥ 2	34 (9.6)	74 (26.7)	8 (8.0)	21 (29.6)	16 (23.5)	4 (11.1)	14 (13.5)	22 (34.4)	20 (25.3)	213 (18.5)
Comorbidities and pathologies at risk of FN^†^, *n* (%)	40 (11.2)	121 (43.2)	22 (21.4)	20 (27.8)	14 (19.4)	7 (17.9)	23 (21.9)	24 (36.4)	32 (39.5)	303 (25.8)
Stage of tumour at inclusion, *n* (%)										
I–IIIb	261 (73.3)	82 (29.3)	23 (22.3)	13 (18.1)	31 (43.1)	9 (23.1)	52 (49.5)	2 (3.0)	25 (30.9)	498 (42.4)
IV	95 (26.7)	198 (70.7)	80 (77.7)	59 (81.9)	41 (56.9)	30 (76.9)	53 (50.5)	64 (97.0)	56 (69.1)	676 (57.6)
Prior G-CSF, n^*^ (%)	43 (12.1)	46 (16.4)	5 (4.9)	7 (9.7)	14 (19.4)	7 (17.9)	12 (11.4)	13 (19.7)	23 (28.4)	170 (14.5)
Prior radiotherapy, n^*^ (%)	81 (22.8)	77 (27.5)	19 (18.4)	10 (13.9)	9 (12.5)	18 (46.2)	44 (41.9)	35 (53.0)	14 (17.3)	307 (26.1)
Prior CT, n^*^ (%)	107 (30.1)	105 (37.5)	47 (45.6)	29 (40.3)	37 (51.4)	17 (43.6)	35 (33.3)	51 (77.3)	26 (32.1)	454 (38.7)
Prior severe neutropenia, n^*^ (%)	39 (11.0)	27 (9.6)	8 (7.8)	9 (12.5)	11 (15.3)	1 (2.6)	4 (3.8)	2 (3.0)	8 (9.9)	109 (9.3)
Grade 3	14 (35.9)	18 (66.7)	4 (50.0)	3 (33.3)	6 (54.5)	1 (100)	2 (50.0)	0 (0)	1 (12.5)	49 (45.0)
Grade 4	23 (59.0)	9 (33.3)	3 (37.5)	5 (55.6)	5 (45.5)	0 (0)	1 (25.0)	1 (50.0)	8 (100)	55 (50.5)
Febrile^‡^	11 (28.2)	10 (37.0)	3 (37.5)	5 (55.6)	2 (18.2)	0 (0)	1 (25.0)	2 (100)	5 (62.5)	39 (35.8)

^*^Some patients had missing data

^†^Hepatic, renal, cardiovascular, respiratory or other comorbidity (alcoholism, diabetes, hypercholesterolaemia or smoking)

^‡^Febrile neutropenia was defined according to the EORTC guidelines

^§^Other ST cohorts include urological outside prostate, sarcoma, thymic carcinoma, skin, bone, unknown primary, multiple independent sites

*CT* chemotherapy, *ECOG PS* Eastern Cooperative Oncology Group performance score, *FN* febrile neutropenia, *G-CSF* granulocyte colony-stimulating factor, *GI* gastrointestinal, *GYN* gynaecological, *SD* standard deviation, *ST* solid tumour

At inclusion, 9.3% (*n* = 109) of patients had previously experienced at least one episode of severe neutropenia, which was predominantly a single episode (89%, *n* = 97), and occurred a mean of 2.7 ± 5 months before study inclusion. Prior episodes of FN were reported for 35.8% of these patients (*n* = 39), with variations according to tumour type.

The proportion of patients who had previously received CT (38.7%, *n* = 454) varied across tumour types, from 30.1% (*n* = 107) in breast cancer to 77.3% (*n* = 51) in prostate cancer. G-CSF therapy had previously been given to 14.5% of ST patients (*n* = 170).

CT planned at inclusion varied depending on the type of the tumour (Table 2). A detailed table of CT therapies planned for patients with ST is shown in Additional file 1: Table S2. The majority of CT regimens planned for breast cancer patients were anthracycline-based CT regimens such as cyclophosphamide/epirubicin/fluorouracil (FEC) +/− sequential docetaxel; for lung cancer were platinum based, mainly carboplatin or cisplatin, in combination with etoposide or paclitaxel or pemetrexed; for digestive cancers were mainly polytherapies with 5-fluorouracil (5-FU), such as 5-FU/l-folinic acid/d,l-folinic acid/irinotecan (FOLFIRI), 5-FU/leucovorin/irinotecan/oxaliplatin (FOLFIRINOX), and 5-FU/folinic acid/oxaliplatin (FOLFOX); and for ovarian cancer were carboplatin regimens, mainly in combination with paclitaxel, which was also the case for non-ovarian gynaecological cancers. Two-thirds of the regimens planned for patients with head and neck cancer were cisplatin based (5-FU/docetaxel/cisplatin [TPF] was most common) and 21.9% were carboplatin based; for prostate cancer most regimens were classed as non-platinum based, being largely docetaxel (first-line metastatic) or cabazitaxel (second- or third-line metastatic). These regimens are categorised as high or intermediate FN risk in EORTC guidelines except for FOLFOX and carboplatin regimens, which are low risk (< 10%), and docetaxel and cabazitaxel, which are not included in the EORTC risk rating but classed as intermediate risk in NCCN guidelines [10].

**Table 2:** Chemotherapy planned at inclusion for the ST cohort (analysis population, *N* = 1174)^*^

Planned CT regimen, *n* (%)	Breast (*n* = 356)	Lung (*n* = 280)	GI: colorectal (*n* = 103)	GI: non-colorectal (*n* = 72)	GYN: ovarian (*n* = 72)	GYN: non-ovarian (*n* = 39)	Head & neck (*n* = 105)	Prostate (*n* = 66)	Other^†^ (*n* = 81)	Total (*N* = 1174)
Adjuvant/neoadjuvant	260 (73.0)	47 (16.8)	22 (21.4)	12 (16.7)	28 (38.9)	7 (17.9)	39 (37.1)	2 (3.0)	23 (28.4)	440 (37.5)
Metastatic	95 (26.7)	198 (70.7)	80 (77.7)	59 (81.9)	41 (56.9)	30 (76.9)	53 (50.5)	64 (97.0)	56 (69.1)	676 (57.6)
Other	1 (0.3)	35 (12.5)	1 (1.0)	1 (1.4)	3 (4.2)	2 (5.1)	13 (12.4)	0 (0)	2 (2.5)	58 (4.9)
First-line	249 (69.9)	173 (62.2)	56 (54.4)	43 (59.7)	35 (48.6)	22 (56.4)	70 (66.7)	15 (22.7)	55 (67.9)	718 (61.3)
≥ Second-line	107 (30.1)	105 (37.8)	47 (45.6)	29 (40.3)	37 (51.4)	17 (43.6)	35 (33.3)	51 (77.3)	26 (32.1)	454 (38.7)
Missing data	0	2	0	0	0	0	0	0	0	2
Anthracyclines	227 (63.8)	–	2 (1.1)		–	–	–	–	–	–
Taxanes (w/o platinum)	82 (23.0)	36 (12.9)	–		–	–	–	–	1 (1.2)	–
Platinum (carboplatin or cisplatin)	16 (4.5)	219 (78.2)	–		–	30 (76.9)	–	4 (6.1)	–	–
No platinum (any cytotoxic regimen)	–	–	–		–	–	–	62 (93.9)	–	–
Bi or tri-therapy with 5-FU	–	–	143 (81.7)		–	–	–	–	–	–
Carboplatin (alone or combined)	–	–	–		56 (77.8)	–	23 (21.9)	–	25 (30.9)	–
Cisplatin (alone or combined)	–	–	–		4 (5.6)	–	70 (66.7)	–	35 (43.2)	–
Others	31 (8.7)	25 (8.9)	30 (17.1)		12 (16.7)	9 (23.1)	12 (11.4)	–	20 (24.7)	–

^*^Some patients had missing data

^†^Other ST cohorts include urological outside prostate, sarcoma, thymic carcinoma, skin, bone, unknown primary, multiple independent sites

*CT* chemotherapy, *5-FU* 5-fluorouracil, *GI* gastrointestinal, *GYN* gynaecological, *ST* solid tumour

### Prescription and use of Zarzio® in routine clinical practice

Zarzio® prescription and use were predominantly in accordance with the label indication for established cytotoxic CT, which states that the recommended dose is 0.5 MIU/kg/day, that the first dose should be administered at least 24h after cytotoxic CT, and that daily dosing should continue until the neutrophil count has recovered (up to 14 days) [19]. Zarzio® treatment was initiated on median Day 4 of the CT cycle at study inclusion, with 64.7% of all patients initiated on Days 2–4 and 21.9% on Days 5–6 (according to the duration of the CT regimen; Additional file 2: Figure S1A). Few patients (0.9%, 10/1174) had Zarzio® on Day 1 of their current CT at study inclusion. Data from the follow-up visit (Additional file 1: Table S3) matched data from study inclusion, showing that this pattern of use did not change during the study.

The median duration of planned Zarzio® treatment was 5 days for all tumour subgroups and no patients were treated for > 14 days (Additional file 1: Table S3; Additional file 2: Figure S1B). Regarding CT cycle duration, 9.8% of patients had 14-day cycles, 67.7% had 21-day cycles, and 6.4% had 28-day cycles. Around 6.5% of patients had a cycle duration of either ≥ 28 days or unspecified, respectively. Patients with GI cancer were more likely to have 14-day or 15-day cycles for their CT regimen (80.6% colorectal, 59.7% non-colorectal GI cancer).

**Fig. 2:**
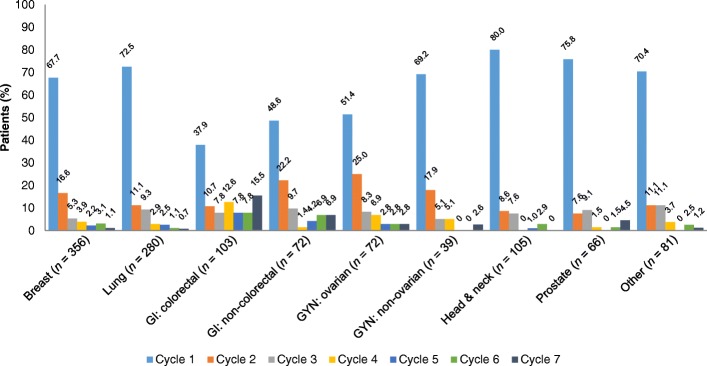
Cycle of CT protocol at study inclusion (*n* = 1174). CT, chemotherapy; GI, gastrointestinal; GYN, gynaecological

With the exception of patients with GI tumours, the majority of the ST analysis population (65.8%, *n* = 773/1174) were in Cycle 1 of their current CT at inclusion, when their treatment with Zarzio® was initiated, and thus were receiving primary prophylaxis (Fig. 2). Patients with ovarian or non-colorectal GI cancers received primary or secondary prophylaxis equally.

In the overall ST cohort, Zarzio® was administered for a median of four CT cycles (three for head and neck cancer, three for lung cancer, five for GI cancers), although the mean number of cycles varied depending on tumour type, stage of disease and CT protocol (Additional file 1: Table S3). Treatment stayed constant for subsequent cycles in nearly all patients (91.8%, *n* = 1047/1141); in the 94 patients who had their treatment modified, a change in treatment duration was the most common modification (77.7%, *n* = 73).

During the course of the study, 39.4% (*n* = 449) of patients stopped Zarzio® treatment after a median of three cycles. The main reason was CT cessation (74.6%, *n* = 335/449), which included reaching the end of the planned protocol (43.9%, *n* = 197/449; Additional file 2: Figure S1C).

### Factors linked to initiation of primary prophylaxis

By definition, prophylaxis with G-CSF was considered primary if occurring during Cycle 1 of the current CT course at study inclusion, regardless of the intended regimen and day of Zarzio® initiation. Zarzio® was prescribed as primary prophylaxis in the majority of patients (65.8%, *n* = 773), though the proportion varied depending on the tumour type (Fig. 2), owing to the CT protocols and line of treatment common to each type.

Initiation of Zarzio® as primary prophylaxis as opposed to secondary prophylaxis or treatment was similar, regardless of whether planned CT was in the adjuvant/neoadjuvant setting or provided as therapy for metastatic disease. Overall, 38.6% of patients initiating Zarzio® as primary prophylaxis had adjuvant/neoadjuvant CT planned, compared with 35.4% initiating Zarzio® in Cycle ≥ 2; similar percentages were observed for patients with CT planned for metastatic disease (55.9% compared with 60.8%).

Patients prescribed primary prophylaxis were similar to the overall ST cohort, being 53.9% female, and having a mean age of 62.2 ± 11.8 years, advanced tumours (55.9% with stage IV tumour), a good performance status (81.5% Eastern Cooperative Oncology Group performance score [ECOG PS] < 2), and few prior episodes of severe neutropenia (3.6%, *n* = 28/773) or FN (1.6%, *n* = 12/773). In patients given primary prophylaxis, clinicians were significantly more likely to prescribe Zarzio® for reasons of concomitant pathologies (23.1% vs 10.9%, *p* < 0.0001), and poor functional or nutritional status (23.8% vs 16.2%, *p* < 0.01), compared with those given Zarzio® in subsequent cycles (Table 3). Patients assessed as high FN risk category (≥ 20%) were significantly more likely to be given Zarzio® as primary prophylaxis (*p <* 0.0001; Table 3).

**Table 3:** Reasons for treatment with Zarzio®: clinician assessment and Zarzio® treatment in patients given primary vs secondary/curative prophylaxis (analysis population, *N* = 1174)

	Breast (*n* = 356)	Lung (*n* = 280)	GI: colorectal (*n* = 103)	GI: non-colorectal (*n* = 72)	GYN: ovarian (*n* = 72)	GYN: non-ovarian (*n* = 39)	Head & neck (*n* = 105)	Prostate (*n* = 66)	Other^‡^ (*n* = 81)	Total (*N* = 1174)	Primary prophylaxis^†^(*n* = 772)	Secondary/curative prophylaxis (*n* = 402)	*p* value^§^
FN risk category according to clinician, *n* (%)													
≥ 20%	133 (37.3)	92 (32.8)	24 (23.3)	24 (33.3)	27 (37.5)	14 (35.9)	49 (46.7)	29 (43.9)	43 (53.1)	435 (37.1)	321 (41.6)	114 (28.4)	<0.0001
10–20% with risk factors	174 (48.9)	162 (57.9)	60 (58.3)	39 (54.2)	43 (59.7)	23 (59.0)	45 (42.8)	32 (48.5)	34 (42.0)	612 (52.1)	389 (50.4)	223 (55.5)	
< 10%	49 (13.8)	26 (9.3)	19 (18.4)	9 (12.5)	2 (2.8)	2 (5.1)	11 (10.5)	5 (7.6)	4 (4.9)	127 (10.8)	62 (8.0)	65 (16.2)	
Reason for G-CSF prescription, *n* (%)^*^													
Elderly patient	65 (18.3)	66 (23.6)	34 (33.0)	18 (25.0)	27 (37.5)	12 (30.8)	19 (18.1)	27 (40.9)	19 (23.5)	287 (24.4)	203 (26.3)	84 (20.9)	0.04
Poor functional or nutritional status	35 (9.8)	78 (27.9)	17 (16.5)	21 (29.2)	15 (20.8)	4 (10.3)	45 (42.9)	13 (19.7)	21 (25.9)	249 (21.2)	184 (23.8)	65 (16.2)	<0.01
Low leucocyte levels	45 (12.6)	45 (16.1)	52 (50.5)	25 (34.7)	18 (25.0)	4 (10.3)	15 (14.3)	12 (18.2)	10 (12.3)	226 (19.3)	45 (12.6)	181 (45.0)	<0.0001
Concomitant pathologies	26 (7.3)	99 (35.4)	15 (14.6)	14 (19.4)	9 (12.5)	6 (15.4)	20 (19.0)	12 (18.2)	21 (25.9)	222 (18.9)	178 (23.1)	44 (10.9)	<0.0001
Low haemoglobin levels	3 (0.8)	22 (7.9)	7 (6.8)	10 (13.9)	10 (13.9)	2 (5.1)	2 (1.9)	12 (18.2)	8 (9.9)	76 (6.5)	38 (4.9)	38 (9.5)	<0.01
Prior FN	19 (5.3)	13 (4.6)	3 (2.9)	4 (5.6)	9 (12.5)	0 (0)	2 (1.9)	1 (1.5)	4 (4.9)	55 (4.7)	24 (3.1)	31 (7.7)	<0.001
FN in previous CT cycle	11 (3.1)	9 (3.2)	5 (4.9)	4 (5.6)	1 (1.4)	0 (0)	1 (1.0)	1 (1.5)	5 (6.2)	37 (3.2)	7 (0.9)	30 (7.5)	<0.0001
Prophylactic antibiotic	0 (0)	5 (1.8)	1 (1.0)	1 (1.4)	0 (0)	0 (0)	1 (1.0)	1 (1.5)	0 (0)	9 (0.8)	4 (0.5)	5 (1.2)	0.29^ǁ^
Other reason^**^	219 (61.5)	106 (37.9)	31 (30.1)	23 (31.9)	31 (43.1)	20 (51.3)	40 (38.1)	33 (50.0)	37 (45.7)	540 (46.0)	391 (50.6)	149 (37.1)	<0.0001

^*^Multiple reasons could be given

^†^Primary prophylaxis for FN defined as G-CSF prophylaxis introduced during the first cycle of the current CT regimen.

^‡^Other ST cohorts include urological outside prostate, sarcoma, thymic carcinoma, skin, bone, unknown primary, multiple independent sites.

^§^Univariate statistical test

^ǁ^Fisher test

^**^Other reasons for G-CSF prescription include previous CT treatment, toxicity to CT, maintaining dose intensity, neutropenia, heavily pre-treated patients and prophylaxis

*CT* chemotherapy, *FN* febrile neutropenia, *G-CSF* granulocyte colony-stimulating factor, *GI* gastrointestinal, *GYN* gynaecological

Patients who had Zarzio® initiated in Cycle ≥ 2 were more likely (*p <* 0.0001) to have had a history of severe or febrile neutropenia at baseline (20.2%, *n* = 81/401, and 6.7%, *n* = 27/401, of patients, respectively). Compared with those receiving primary prophylaxis, clinicians were more likely to initiate Zarzio® in Cycle ≥ 2 for reasons of FN history (7.7% vs 3.1%, *p <* 0.01), FN in the preceding cycle of the current CT (7.5% vs 0.9%, *p <* 0.0001), low leucocyte levels (45.0% vs 12.6%, *p <* 0.0001), and low haemoglobin levels (9.5% vs 4.9%, *p <* 0.01; Table 3).

### Use of Zarzio® in relation to guideline recommendations

The patient profile of the ST cohort was assessed in terms of guidelines for FN risk due to planned CT regimen and for patient-related risk factors (primarily EORTC recommendations [10], with reference to NCCN 2016 [12] and ASCO 2015 [13] guidelines where the EORTC recommendation did not provide a risk category for a particular regimen). Overall, few patients given Zarzio® had planned CT regimens associated with FN risk < 10%, in line with EORTC recommendations. Patients with ovarian cancer were the exception, where 38.9% of those receiving Zarzio® had a planned CT regimen associated with a low risk of FN. A substantial proportion of patients had CT regimens for which a risk category was not given in the guidelines, though this varied between tumour types (Table 4). Almost all patients (99.1%, *n* = 1160/1170 patients with evaluable data) had at least one of the patient-related risk factors for FN listed in the EORTC recommendations (Table 4), as did those whose CT regimen was of unknown FN risk (99.6%, *n* = 536/538). These included advanced tumour stage (stage III or IV; 81.9%, *n* = 961) and age over 65 years (41.7%, *n* = 489). Very few patients overall (3.3%, *n* = 39/1174) had the risk factor of severe FN episodes prior to study inclusion. Patients whose CT regimen was in the EORTC low-risk category also had the EORTC-specified patient-related factors for increased FN risk of advanced tumour stage (87.5%), age of over 65 years (48.2%), and low haemoglobin levels (58.8%).

**Table 4:** Febrile neutropenia risk of CT regimen and additional risk factors per EORTC guidelines (analysis population)

	Breast (*n* = 356)	Lung (*n* = 280)	GI: colorectal (*n* = 103)	GI: non-colorectal (*n* = 72)	GYN: ovarian (*n* = 72)	GYN: non-ovarian (*n* = 39)	Head & neck (*n* = 105)	Prostate (*n* = 66)	Other^‡^ (*n* = 81)	Total (*n* = 1174)
Patients with CT regimen in EORTC FN risk category, *n* (%)										
Low (< 10%)	0 (0)	15 (5.4)	0 (0)	0 (0)	28 (38.9)	0 (0)	1 (1)	0 (0)	12 (14.8)	56 (4.8)
Intermediate (10–20%)	253 (71.1)	139 (49.6)	43 (41.7)	30 (41.7)	2 (2.8)	0 (0)	5 (4.8)	40 (60.6)	1 (1.2)	513 (43.7)
High (≥ 20%)	12 (3.4)	30 (10.7)	0 (0)	1 (1.4)	2 (2.8)	0 (0)	0 (0)	0 (0)	20 (24.7)	65 (5.5)
Unknown	91 (25.6)	96 (34.3)	60 (58.3)	41 (56.9)	40 (55.6)	39 (100)	99 (94.3)	26 (39.4)	48 (59.3)	540 (46.0)
Patients with patient-related EORTC risk factor, *n* (%)										
Age > 65 years	94 (26.4)	120 (42.9)	52 (50.5)	34 (47.2)	42 (58.3)	24 (61.5)	32 (30.5)	53 (80.3)	38 (46.9)	489 (41.7)
Female gender	351 (98.6)	87 (31.1)	45 (43.7)	26 (36.1)	72 (100)	39 (100)	15 (14.3)	0 (0)	12 (14.8)	647 (55.1)
Advanced tumour: stage III or IV	199 (55.9)	265 (94.6)	102 (99.0)	66 (91.7)	65 (90.3)	34 (87.2)	90 (85.7)	65 (98.5)	75 (92.6)	961 (81.9)
ECOG PS ≥ 2^*^	34 (9.6)	74 (26.7)	8 (8.0)	21 (29.6)	16 (23.5)	4 (11.1)	14 (13.5)	22 (34.4)	20 (25.3)	213 (18.5)
Haemoglobin level < 12 g/dL^*^	88 (27.7)	120 (44.8)	44 (44.0)	39 (56.5)	43 (64.2)	25 (67.6)	31 (36.0)	38 (62.3)	42 (53.2)	470 (43.3)
Prior episode of FN	11 (3.1)	10 (3.6)	3 (2.9)	5 (6.9)	2 (2.8)	0 (0)	1 (1.0)	2 (3.0)	5 (6.2)	39 (3.3)
Concomitant pathologies with increased risk^†^	11 (3.1)	56 (20.0)	5 (4.9)	4 (5.6)	4 (5.6)	4 (10.3)	9 (8.6)	9 (13.6)	19 (23.5)	121 (10.3)
No G-CSF use before inclusion	318 (89.3)	270 (96.4)	90 (87.4)	68 (94.4)	64 (88.9)	34 (87.2)	99 (94.3)	60 (90.9)	72 (88.9)	1075 (91.6)

^*^Some patients had missing data

^†^Liver, renal or cardiovascular disease, according to EORTC definition

^‡^Other ST cohorts include urological outside prostate, sarcoma, thymic carcinoma, skin, bone, unknown primary, and multiple independent sites

*CT* chemotherapy, *ECOG PS* Eastern Cooperative Oncology Group Performance Status, *EORTC* European Organisation for Research and Treatment of Cancer, *FN* febrile neutropenia, *G-CSF*, granulocyte colony-stimulating factor, *GI* gastrointestinal, *GYN* gynaecological, *ST* solid tumour

Clinicians’ reasons for giving Zarzio® and their assessment of the FN risk of the CT regimen planned for the patient are shown in Table 3. In the analysis population, clinicians classed most of their patients as having an intermediate or high risk of FN due to their CT regimen, with only 10.8% of patients (*n* = 127) classed as low risk (< 10%). Clinicians’ reasons for prescribing Zarzio® were mostly old age, poor functional or nutritional status, low leucocyte levels, and concomitant pathologies. Clinicians’ reasons for prescribing Zarzio® to patients whose CT regimen FN risk was unknown were similar (old age [27.2%], poor functional or nutritional status [24.8%], low leucocyte levels [25.4%] and concomitant pathologies [20.2%]). Clinicians’ reasons for prescribing G-CSF to patients with planned CT regimens with a low FN risk were mainly old age (30.4%), poor functional or nutritional status (21.4%), concomitant pathologies (19.6%), low leucocyte levels (19.6%) and low haemoglobin levels (12.5%), appear to be in response to the EORTC-specified patient-related factors for increased risk of FN. Other reasons for prescribing G-CSF included previous CT treatment, toxicity to CT, maintaining dose intensity, neutropenia, heavily pre-treated patients and prophylaxis.

The extent of agreement between the EORTC guideline assessment of FN risk due to the planned CT regimen and that assessed by the clinicians in this study is shown in Table 5. While the majority of clinicians’ assessments were aligned to the guideline risk categories given for the CT regimens, differences were noticeable. Few patients (*n* = 56/1174) had CT regimens categorised in guidelines as low FN risk, yet clinicians mostly assessed these as having intermediate or high risk of FN (64.3% and 32.1%, respectively). Conversely, some patients in each of the high, intermediate or unknown risk categories according to their CT regimen were assessed as low risk by the clinicians (6.2%, 12.3%, and 10.7%, respectively). Of patients with CT regimens in the intermediate-risk category (*n* = 513/1174), half of these (50.3%) were classed by clinicians as having intermediate risk and 37.4% as high risk of FN. Few patients had CT regimens in the high-risk category (*n* = 65/1174), and the greatest agreement between clinician assessment and guideline category was seen in this group, with 64.6% assessed as high FN risk and 29.2% as intermediate risk. Patients with CT regimens whose guideline FN risk category was unknown were the largest group (*n* = 540/1174), with clinicians assessing these as mostly high (33.9%) or intermediate (55.4%) risk of FN.

**Table 5:** Agreement of clinicians’ assessment of FN risk category with EORTC recommendations for CT regimens (*N* = 1174)

FN risk category of CT regimen assessed by clinician	Patients in FN risk category assessed according to recommendations, *n* (%)			
	< 10% (*n* = 56)	10–20% (*n* = 513)	≥ 20% (*n* = 65)	Unknown (*n* = 540)
< 10%	**2 (3.6)**	63 (12.3)	4 (6.2)	58 (10.7)
10–20% (with patient risk factors)	36 (64.3)	**258 (50.3)**	19 (29.2)	299 (55.4)
≥ 20%	18 (32.1)	192 (37.4)	**42 (64.6)**	183 (33.9)

Bold numbers indicate patients where the clinicians’ assessment of FN risk category agrees with the EORTC-recommended FN risk category

*CT* chemotherapy, *EORTC* European Organisation for Research and Treatment of Cancer, *FN* febrile neutropenia

### Rates of neutropenia

**Fig. 3:**
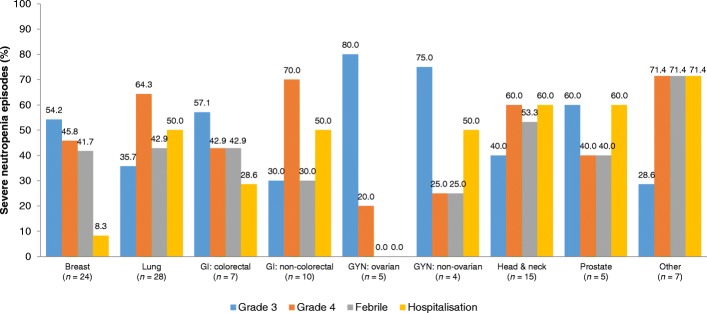
Characteristics of severe (grade 3–4) neutropenia episodes (*n* = 105) in the ST population during the study. GI, gastrointestinal; GYN, gynaecological

Severe neutropenia presented in 7.6% (*n* = 87) of patients followed up during the study (Fig. 3), and FN presented in 3.5% (*n* = 40). Incidence of FN varied between tumour types, from 7.7% (*n* = 8) of patients with head and neck cancer to 0% with ovarian cancers.

Patients who presented with FN predominantly had advanced (stage ≥ III) cancer at study entry (72.5%, *n* = 29), with good functional performance (80.0%, *n* = 32, ECOG PS of 0–1), were aged 65 years or younger (75.0%, *n* = 30), and had no previous episodes of severe neutropenia (80.0%, *n* = 32) or FN (85.0%, *n* = 34).

### Safety

Throughout the study, 62 AEs associated with Zarzio® occurred in 3.4% (*n* = 39) of all patients (Additional file 1: Table S4). Adverse events were mild (36.4%, *n* = 16), moderate (50.0%, *n* = 22) or severe (13.6%, *n* = 6); 18 AEs were not described. Fourteen AEs (22.6%) were considered serious, and 48 (77.4%) were considered non-serious. Musculoskeletal and connective tissue disorders were the most commonly reported per system organ class of AEs (41.9%, *n* = 26), followed by general disorders and administration site conditions (14.5%, *n* = 9), blood and lymphatic system disorders (12.9%, *n* = 8), and GI disorders (9.7%, *n* = 6). Discontinuation of G-CSF treatment due to G-CSF toxicity was observed in less than 5% of patients.

## Discussion

Given that biosimilar G-CSFs for treating CT-induced neutropenia are well established in Europe, the ZOHé study aimed to investigate how the biosimilar filgrastim Zarzio® is currently used in routine clinical practice by haematologists and oncologists in France. In this large prospective study of current practice, prescription and use of Zarzio® were found to be predominantly compliant with the label indication. For most patients, actual Zarzio® use did not change from that planned at initiation. Few AEs assessed as related to Zarzio® were reported during the study and no unexpected AEs were seen, confirming its safety profile.

Zarzio® use in this study is largely in keeping with EORTC guidelines for G-CSF prophylaxis for neutropenia, with almost all patients (99.1% of evaluable patients) having at least one patient-related factor for increased risk of FN [10]. At study entry, the patients were mostly elderly (mean age 62.3 years, 27.4% ≥ 70 years old), with metastatic disease (57.6% stage IV) but a good performance status (97.5% ECOG PS 0–1).

Primary prophylaxis was the main indication (65.8% of patients), though this varied depending on tumour type, most likely due to the risks of FN inherent to different tumour types and their treatment regimens. Few patients had a history of prior severe FN, and these were more likely to be given Zarzio® as secondary prophylaxis. Presence of comorbidities or pathologies that increase FN risk and an overall high FN risk category of the CT were identified as the main factors driving the clinicians’ decision to initiate primary prophylaxis with Zarzio®. This suggests that in routine practice clinicians are generally using Zarzio® in accordance with the European guidelines.

In ZOHé, nearly half of the ST patients (46.0%) received CT regimens that were not given an FN risk category in EORTC or other guidelines (ASCO and NCCN [12, 13]), highlighting the need for updated recommendations and inclusion of guidance as to the use of G-CSF therapy together with novel chemotherapeutic agents, for which identification of FN risk should be performed in early phases of development. Furthermore, most guidelines are based on clinical trials and the specially chosen patient populations therein, which do not necessarily reflect patients encountered in routine clinical practice. Thus, it may be more appropriate for clinicians to base their treatment decisions around the use of G-CSF therapy on patient-specific risk factors rather than guideline recommendations. In the ZOHé study, only 41% of patients receiving primary prophylaxis with biosimilar filgrastim had an FN risk according to the clinician of > 20%, a threshold that has historically been set to 20% in relation to economic analyses at the time. Patients who received G-CSF as secondary prophylaxis had a lower rate of underlying risk factors and a higher rate of prior neutropenia, driving the decision for clinical use. In all other cases, clinicians’ decisions to prescribe G-CSF already appeared to be based on patient-related risk factors rather than toxicity of the CT regimen. In real-world practice, as reported in the ZOHé study, patient-related risk factors are more likely to be a driver of the clinicians’ decision to initiate Zarzio® treatment than the risk category of the CT regimen.

Other factors impacting the effectiveness of G-CSF therapy are the starting day after CT and the duration of G-CSF use. An Italian study assessed clinician adherence to timing of G-CSF treatment post-CT and found that G-CSF prophylaxis was frequently administered in a manner that is not supported by evidence-based guidelines [29], which recommend daily G-CSF administration within 24–72 h post-CT. Type of G-CSF regimen and pattern of use has also been associated with differential FN treatment outcomes in a Spanish population with non-myeloid tumours, where daily use of filgrastim for only 5–6 days may have impacted treatment benefit negatively in terms of FN prophylaxis [25]. Furthermore, overuse of G-CSF in patients without additional risk factors and variability in adherence to guidelines depending on the tumour type treated were previously reported [30]. Therefore, it is essential to not only provide updated treatment guidelines addressing these variations in practice, but also educate the oncology community about correct use of therapies and highlight physician overestimation of their adherence to the guidelines [30]. Clinicians assessed in this study initiated Zarzio® on median Day 4 of the CT cycle with 64.7% of all patients initiated on Days 2–4 and 21.9% on Days 5–6 (partially due to CT duration of 1, 2 or 3 days), potentially leading to poor treatment outcomes. It was further shown that, once this treatment pattern was established, it rarely changed throughout the study duration.

Of note is that clinicians tended to assess a higher FN risk category for a given CT regimen than that documented in the guidelines. Weak correlations between physician-assessed risk and model-predicted risk for FN have been noted elsewhere [31]. Given the multiple patient-, disease- and treatment-specific factors for individual FN risk, precise and consistent FN risk assessment is essential for the appropriate use of G-CSF to optimise patient care. The ZOHé study emphasises the relatively poor correlation between the clinicians’ assessment of FN and the risk as established in the literature. In addition, nearly half of the regimens used did not have an established FN risk reported, reflecting the need for better reporting of FN rates from clinical trials.

Regarding the 10.8% of patients who were given Zarzio® despite being categorised as low FN risk, this ‘over prophylaxis’ was also seen in the MONITOR-GCSF study of Zarzio® treatment patterns and FN incidence across 12 countries, including France [23]. Gascón et al. reported that 26.0% of patients were given Zarzio® as primary prophylaxis, despite being below the risk threshold according to EORTC guidelines [23]. The increased availability of G-CSF biosimilars may be a factor in such ‘over prophylaxis’ in a small subset of patients not usually eligible for Zarzio® treatment. While earlier studies found that G-CSF prophylaxis for CT-induced neutropenia was underused in European real-world practice [25, 26], more recent studies suggest that the widespread availability and reduced cost of biosimilar G-CSFs are leading to increased use in clinical practice [15, 21, 27]. EORTC criteria might be re-evaluated by pooled analysis of real-life trials with calculation of ‘over prophylaxis’ in patients with FN risk < 10%.

The strength of this prospective study includes the large sample size (1179 patients with ST, 125 centres), enabling successful analysis of all planned endpoints, although data were missing or incomplete for a number of patients in the study. Limitations of the study include selection bias, recall bias and the need to pool data. Selection bias is inherent to the voluntary participation in a prospective study, and was addressed by recruiting physicians so that the sample of investigators reflected the practice of physicians throughout France, and by including patients sequentially and limiting their number to 35 per site. Comparison of details collected for participating and non-participating physicians showed little difference except in gender balance, and the diversity of enrolled patient profiles suggests that investigator selection of patients was unlikely. The primary study objective of describing Zarzio® use across routine clinical practice in a variety of conditions limited the overall interpretations of the individual clinical situations, as it was necessary to group CT protocols by category and treatment stage to assess the results. Furthermore, although this study provides an insight into the treatment patterns with filgrastim, it does not address whether these changing patterns of use of G-CSF relate to the biosimilar per se or simply changing patterns of G-CSF use in general.

## Conclusions

During this large prospective study of Zarzio® use in routine clinical practice across France in patients undergoing CT for ST, conditions of Zarzio® use were relatively standardised, regardless of tumour type and variations in patient characteristics, and were compliant with the label indication. Zarzio® was most commonly given as primary prophylaxis, during the first cycle of CT, and treatment lasted for a median of four cycles. Incidence of FN and AEs was low and in keeping with previous assessments of Zarzio®. Use of Zarzio® in routine clinical practice for prophylaxis of CT-induced neutropenia is generally in line with EORTC guidelines; however, overuse of prophylaxis was observed in a small group of patients classed with a FN risk of < 10%, in which G-CSF use may not necessarily be recommended. Most patients in this study were receiving intermediate- or high-risk chemotherapy regimens for FN and > 90% of patients had patient risk factors, thus representing a large patient population eligible for treatment with Zarzio®. Some misalignment between physicians’ assessment of FN risk and EORTC guidelines risk assessment for individual CT regimens was seen. Many CT regimens are not listed for their FN risk in guideline recommendations, but patient-related risk factors are a stronger driver of clinicians’ decision to initiate Zarzio® treatment. G-CSF prophylaxis in real-world practice is increased compared with earlier studies reporting underuse. From this study and others, optimised recommendations should improve good clinical practice in oncology.

## Additional files

Additional file 1: **Table S1.** Reasons for premature withdrawal (*n* = 294). **Table S2.** Most common planned CT therapies for patients with ST (analysis population; *n* = 1174). **Table S3.** Characteristics of treatment with Zarzio® during the study in patients with follow up (*n* = 1141). **Table S4**. Summary of adverse events (*n* = 62) experienced by ST patients during the study. (DOCX 22 kb)

Additional file 2: **Figure S1.** Characteristics of Zarzio®. **A**: First day in the CT cycle that Zarzio is administered. **B**: Duration of Zarzio® administration. **C**: Reasons given for stopping Zarzio® before study end.^†^ Data on the use of Zarzio® in routine clinical practice are for the patients with data collected at follow up (*n* = 1141). *Zarzio® begun the same day as the CT cycle. ^†^Multiple reasons could be given. AE, adverse event; CT, chemotherapy; GI, gastrointestinal; GYN, gynaecological; MIU, Million International Units; SD, standard deviation. (DOCX 98 kb)

## Abbreviations

5-FU: 5-fluorouracil
AE: Adverse events
ASCO: American Society of Clinical Oncology
CT: Chemotherapy
ECOG: Eastern Cooperative Oncology Group
EORTC: European Organisation for Research and Treatment of Cancer
ESMO: European Society for Medical Oncology
FEC: Cyclophosphamide/epirubicin/fluorouracil
FEC-D: Cyclophosphamide/epirubicin/fluorouracil/docetaxel
FN: Febrile neutropenia
FOLFIRI: 5-fluorouracil/l-folinic acid/d,l-folinic acid/irinotecan
FOLFIRINOX: 5-fluorouracil/leucovorin/irinotecan/oxaliplatin
FOLFOX: 5-fluorouracil/folinic acid/oxaliplatin
G-CSF: Granulocyte colony-stimulating factor
GI: Gastrointestinal
GYN: Gynaecological
HM: Haematological malignancies
MedDRA: Medical Dictionary for Regulatory Activities
MIU: Million International Units
NCCN: National Comprehensive Cancer Network
SD: Standard deviation
ST: Solid tumours
