# Supplementary material for: Biosimilar filgrastim treatment patterns and prevention of febrile neutropenia: a prospective multicentre study in France in patients with solid tumours (the ZOHé study)
Source: BMC Cancer. 2018 Nov 16;18:1127. doi: 10.1186/s12885-018-4986-1 (PMC6240200; doi:10.1186/s12885-018-4986-1)
Supplement: Supplementary file 1 — Table S1. Reasons for premature withdrawal (n = 294). Table S2. Most common planned CT therapies for patients with ST (analysis population; n = 1174). Table S3. Characteristics of treatment with Zarzio® during the study in patients with follow up (n = 1141). Table S4. Summary of adverse events (n = 62) experienced by ST patients during the study. (DOCX 22 kb) [file 12885_2018_4986_MOESM1_ESM.docx]

**Table S1** Reasons for premature withdrawal (*n* = 294)

| **Reasons** | **Number of patients** | **%** |
| --- | --- | --- |
| Withdrawal of the chemotherapy before 3-month follow up | 139 | 47.3 |
| Withdrawal of Zarzio^®^ before 3-month follow up | 108 | 36.7 |
| Death | 57 | 19.4 |
| Patient lost to follow up | 20 | 6.8 |
| Decision of the patient | 4 | 1.4 |
| Other reason* | 22 | 7.5 |
| Change of therapeutic strategy | 3 | 13.6 |
| Incomplete records | 15 | 68.2 |
| Herceptin maintenance therapy | 1 | 4.5 |
| Orthopaedic surgery scheduled | 1 | 4.5 |
| No premature withdrawal; Zarzio^®^ prescribed until 23 April 2014 | 1 | 4.5 |
| Palliative care | 1 | 4.5 |

*Reasons listed below are given as % of ‘other reason’ group (*n* = 22).

**Table S2** Most common planned CT therapies for patients with ST (analysis population; *n* = 1174)

| **Therapy** | **Breast**  **(*n* = 356)** | **Lung**  **(*n* = 280)** | **GI:**  **colorectal**  **(*n* = 103)** | **GI: non-colorectal**  **(*n* = 72)** | **GYN:**  **ovarian**  **(*n* = 72)** | **GYN:**  **non-ovarian**  **(*n* = 39)** | **Head & neck**  **(*n* = 105)** | **Prostate**  **(*n* = 66)** | **Other**  **(*n* = 81)** | **Total**  **(*n* = 1174)** |
| --- | --- | --- | --- | --- | --- | --- | --- | --- | --- | --- |
| Cabazitaxel | – | – | – | – | – | – | – | 40 (60.6%) | – | 40 (3.4%) |
| Carboplatin etoposide | – | 47 (16.8%) | 1 (1.0%) | 1 (1.4%) | – | – | 1 (1.0%) | 1 (1.5%) | 1 (1.2%) | 52 (4.4%) |
| Carboplatin paclitaxel or carboplatin paclitaxel bevacizumab | 2 (0.6%) | 31 (11.1%) | – | – | 34 (47.2%) | 22 (56.4%) | 4 (3.8%) | 1 (1.5%) | 4 (4.9%) | 100 (8.5%) |
| Docetaxel or docetaxel trastuzumab or docetaxel trastuzumab pertuzumab | 30 (8.4%) | 26 (9.3%) | – | 2 (2.8%) | 1 (1.4%) | – | – | 17 (25.8%) | – | 76 (6.5%) |
| Docetaxel platine 5-FU | – | – | – | 1 (1.4%) | – | – | 36 (34.3%) | – | – | 37 (3.2%) |
| Fec (5FU epirubicin cyclophosphamide) | 80 (22.5%) | – | – | – | – | – | – | – | – | 80 (6.8%) |
| Fec docetaxel or fec docetaxel trastuzumab | 75 (21.1%) | – | – | – | – | – | – | – | – | 75 (6.4%) |
| Folfiri (5FU folinic acid irinotecan) or folfiri aflibercept or folfiri bevacizumab or folfiri trastuzumab | – | – | 38 (36.9%) | 10 (13.9%) | – | – | – | – | – | 48 (4.1%) |
| Folfox (FU folinic acid oxaliplatine) or mfolfox | – | – | 27 (26.2%) | 10 (13.9%) | – | – | – | – | – | 37 (3.2%) |
| Paclitaxel or paclitaxel bevacizumab or paclitaxel trastuzumab or paclitaxel trastuzumab pertuzumab | 21 (5.9%) | 8 (2.9%) | – | 1 (1.4%) | – | 1 (2.6%) | 2 (1.9%) | 1 (1.5%) | – | 34 (2.9%) |
| Others | 148 (41.6%) | 168 (60.0%) | 37 (35.9%) | 47 (65.3%) | 35 (48.6%) | 16 (41.0%) | 62 (59.0%) | 6 (9.1%) | 76 (93.8%) | 595 (50.7%) |
| CT, chemotherapy; ST, solid tumour | | | | | | | | | | |

**Table S3** Characteristics of treatment with Zarzio^®^ during the study in patients with follow up (*n* = 1141)

| **Characteristic** | **Breast**  **(*n* = 349)** | **Lung**  **(*n* = 270)** | **GI:**  **colorectal**  **(*n* = 96)** | | | **GI: non-colorectal**  **(*n* = 69)** | | **GYN:**  **ovarian**  **(*n* = 69)** | **GYN:**  **non-ovarian**  **(*n* = 37)** | **Head & neck**  **(*n* = 104)** | **Prostate**  **(*n* = 66)** | **Other***  **(*n* = 81)** | **Total**  **(*n* = 1141)** |
| --- | --- | --- | --- | --- | --- | --- | --- | --- | --- | --- | --- | --- | --- |
| Number of CT cycles on Zarzio^®^, mean ± SD | 3.8 ± 1.4 | 3.2 ± 1.4 | 4.7 ± 1.6 | 4.4 ± 1.8 | | | 3.7 ± 1.3 | | 3.8 ± 1.4 | 3.2 ± 1.5 | 3.8 ± 1.5 | 3.5 ± 1.3 | 3.7 ± 1.5 |
| **First day of Zarzio^®^ administration per CT cycle** | | | | | | | | | | | | | |
| Mean ± SD | 3.9 ± 2.0 | 4.5 ± 2.1 | 3.7 ± 1.1 | | 4.4 ± 2.1 | | 4.0 ± 2.3 | | 4.1 ± 2.7 | 5.5 ± 2.8 | 3.8 ± 1.5 | 5.6 ± 2.4 | 4.3 ± 2.2 |
| **Duration of Zarzio^®^ administration per CT cycle** | | | | | | | | | | | | | |
| Mean ± SD | 5.2 ± 1.6 | 5.1 ± 1.3 | 4.4 ± 1.1 | | 4.8 ± 1.4 | | 5.2 ± 1.6 | | 5.1 ± 1.6 | 4.8 ± 1.5 | 5.3 ± 1.7 | 5.0 ± 1.4 | 5.0 ± 1.5 |
| **Dose per CT cycle, *n* (%)** | | | | | | | | | | | | | |
| 30 MIU/day | 305 (87.4) | 210 (77.8) | 91 (94.8) | 65 (94.2) | | | 60 (87.0) | | 34 (91.9) | 70 (67.3) | 47 (71.2) | 50 (61.7) | 932 (81.7) |
| > 30≤48 MIU/day | 2 (0.6) | 1 (0.4) | 0 (0) | 0 (0) | | | 0 (0) | | 0 (0) | 3 (2.9) | 1 (1.5) | 0 (0) | 7 (0.6) |
| 48 MIU/day | 42 (12.0) | 59 (21.9) | 5 (5.2) | 4 (5.8) | | | 9 (13.0) | | 3 (8.1) | 31 (29.8) | 18 (27.3) | 31 (38.3) | 202 (17.7) |
| Zarzio^®^ stopped before study end, % | 42.1 | 42.6 | 22.9 | 24.6 | | | 39.1 | | 24.3 | 52.9 | 33.3 | 43.2 | 39.4 |

*Other ST cohorts include urological outside prostate, sarcoma, thymic carcinoma, skin, bone, unknown primary, and multiple independent sites.

AE, adverse event; CT, chemotherapy; GI, gastrointestinal; GYN, gynaecological; MIU, Million International Units; SD, standard deviation

**Table S4** Summary of adverse events (*n* = 62) experienced by ST patients during the study

| **AEs (MedDRA term)** | **AEs, *n* (%)** |
| --- | --- |
| Bone pain | 13 (21.0) |
| Back pain | 7 (11.3) |
| Neutropenia | 4 (6.5) |
| Pain | 4 (6.5) |
| Arthralgia | 3 (4.8) |
| Diarrhoea | 3 (4.8) |
| Fatigue | 2 (3.2) |
| Febrile neutropenia | 2 (3.2) |
| Headache | 2 (3.2) |
| Myalgia | 2 (3.2) |
| Nausea | 2 (3.2) |
| Skin lesion | 2 (3.2) |
| Abdominal pain | 1 (1.6) |
| Aplasia | 1 (1.6) |
| Asthenia | 1 (1.6) |
| Bronchial carcinoma | 1 (1.6) |
| Drug ineffective | 1 (1.6) |
| Dyspnoea | 1 (1.6) |
| Febrile bone marrow aplasia | 1 (1.6) |
| Malignant neoplasm progression | 1 (1.6) |
| Metastatic neoplasm | 1 (1.6) |
| Pain in extremity | 1 (1.6) |
| Pleural effusion | 1 (1.6) |
| Pruritus | 1 (1.6) |
| Pyrexia | 1 (1.6) |
| Thrombocytopenia | 1 (1.6) |
| Urinary tract infection | 1 (1.6) |
| Vasculitis | 1 (1.6) |

AEs with an incidence of ≥1% are shown.

AE, adverse event; MedDRA, Medical Dictionary for Regulatory Activities; ST, solid tumour
