# Supplementary material for: Biosimilar filgrastim treatment patterns and prevention of febrile neutropenia: a prospective multicentre study in France in patients with solid tumours (the ZOHé study)
Source: BMC Cancer. 2018 Nov 16;18:1127. doi: 10.1186/s12885-018-4986-1 (PMC6240200; doi:10.1186/s12885-018-4986-1)
Supplement: Supplementary file 2 — Figure S1. Characteristics of Zarzio®. A: First day in the CT cycle that Zarzio is administered. B: Duration of Zarzio® administration. C: Reasons given for stopping Zarzio® before study end.† Data on the use of Zarzio® in routine clinical practice are for the patients with data collected at follow up (n = 1141). *Zarzio® begun the same day as the CT cycle. †Multiple reasons could be given. AE, adverse event; CT, chemotherapy; GI, gastrointestinal; GYN, gynaecological; MIU, Million International Units; SD, standard deviation. (DOCX 98 kb) [file 12885_2018_4986_MOESM2_ESM.docx]

**A**

**B**

**C**
